# Non-Silent Story on Synonymous Sites in Voltage-Gated Ion Channel Genes

**DOI:** 10.1371/journal.pone.0048541

**Published:** 2012-10-31

**Authors:** Tong Zhou, Eun A. Ko, Wanjun Gu, Inja Lim, Hyoweon Bang, Jae-Hong Ko

**Affiliations:** 1 Institute for Personalized Respiratory Medicine, University of Illinois at Chicago, Chicago, Illinois, United States of America; 2 Section of Pulmonary, Critical Care, Sleep & Allergy, Department of Medicine, University of Illinois at Chicago, Chicago, Illinois, United States of America; 3 Department of Medicine, University of California San Francisco, San Francisco, California, United States of America; 4 Key Laboratory of Child Development and Learning Science of Ministry of Education of China, Southeast University, Nanjing, Jiangsu, China; 5 Department of Physiology, College of Medicine, Chung-Ang University, Seoul, South Korea; University of South Florida College of Medicine, United States of America

## Abstract

Synonymous mutations are usually referred to as “silent”, but increasing evidence shows that they are not neutral in a wide range of organisms. We looked into the relationship between synonymous codon usage bias and residue importance of voltage-gated ion channel proteins in mice, rats, and humans. We tested whether translationally optimal codons are associated with transmembrane or channel-forming regions, i.e., the sites that are particularly likely to be involved in the closing and opening of an ion channel. Our hypothesis is that translationally optimal codons are preferred at the sites within transmembrane domains or channel-forming regions in voltage-gated ion channel genes to avoid mistranslation-induced protein misfolding or loss-of-function. Using the Mantel-Haenszel procedure, which applies to categorical data, we found that translationally optimal codons are more likely to be used at transmembrane residues and the residues involved in channel-forming. We also found that the conservation level at synonymous sites in the transmembrane region is significantly higher than that in the non-transmembrane region. This study provides evidence that synonymous sites in voltage-gated ion channel genes are not neutral. Silent mutations at channel-related sites may lead to dysfunction of the ion channel.

## Introduction

Ion channels are membrane protein complexes that help establish and control the voltage gradient across biological membranes by allowing the flow of ions down their electrochemical gradient. Ion channels play vital roles in diverse cellular processes such as cardiac, skeletal, and smooth muscle contraction, epithelial transport of nutrients and ions, T-cell activation and pancreatic beta-cell insulin release, hormonal secretion, and osmotic regulation of blood pressure [1], [2], [3], [4], [5]. Ion channel dysfunction can have profound physiological effects [6]. Therefore, ion channels are frequently considered as drug targets [7], [8], [9].

The ion channel conformational change between the closed and open states is called gating. Ion channels can be classified by gating, such as the chemical or physical modulator that controls their opening or closing activity. Voltage-gated ion channels open or close depending on the voltage gradient across the plasma membrane. It has been found that the amino acid sequences involved in pore-forming are highly conserved in voltage-gated ion channel proteins [10], [11], [12]. Even a single-site mutation in these regions may lead to a change in channel conductance, voltage dependence, or activity level [6], which suggests that the non-synonymous sites in transmembrane domains of voltage-gated ion channel genes are under stronger purifying selection than the sites in other regions in the same genes [13]. However, the effect of synonymous mutations in voltage-gated ion channel genes is still unknown.

Synonymous mutations (so-called silent mutations) are the change of one base for another in an exon of a gene, but the coded amino acid is not changed. When a synonymous or silent mutation occurs, the change is often assumed to be neutral, meaning that it does not affect the fitness of the individual carrying the new gene to survive and reproduce. However, increasing evidence shows that synonymous mutations are not neutral in a wide range of organisms. For example, selection on synonymous sites has been linked to transcription, splicing, DNA secondary structure, messenger RNA secondary structure and stability, and protein expression [14], [15], [16], [17], [18], [19], [20], [21], [22], [23]. More importantly, selection on synonymous sites for translation with high fidelity has been observed in bacteria, plants, yeast, flies, worms, and even mammals [24], [25], [26], [27].

Translation is an error-prone process [28]. Translation errors occur at frequencies of several misincorporations *per* 10,000 codons translated; precise error rates vary over nearly an order of magnitude among codons [29]. At this error rate, 15% of average-length protein molecules will contain at least one misincorporated amino acid [28]. According to the mistranslation-induced-protein-misfolding hypothesis, selection should prefer high-fidelity codons (optimal codons) at sites at which translation errors are structurally disruptive and lead to protein misfolding, aggregation or dysfunction [30]. For example, the usage of optimal codons was found to be increased in putative zinc-finger and homeodomain regions of transcription factors [24]. Also, optimal codons were reported to be more likely to encode residues in the core of proteins to minimize the misfolding of mistranslated proteins [26].

Here, we investigate whether synonymous codon usage is linked to key residues in voltage-gated ion channel proteins. Specifically, we test whether translationally optimal codons are associated with transmembrane segments or channel-forming regions, i.e., sites that are particularly likely to be involved in the closing and opening of ion channels. Our hypothesis is that translationally optimal codons are preferred at sites within the transmembrane domains or channel-forming regions in voltage-gated ion channel genes. We consider three mammalian organisms: human, rat, and mouse. Using the Mantel-Haenszel procedure, which applies to categorical data, we find that translationally optimal codons are more likely to be used at transmembrane residues and the residues involved in channel-forming. We also find that the conservation level at synonymous sites in transmembrane regions is significantly higher than that in non-transmembrane regions.

**Table 1:** Voltage-gated ion channel genes involved in this study.

Channel type	Gene^a^
Calcium-activated potassium channel	*KCNMA1*, *KCNN1*, *KCNN2*, *KCNN3*, *KCNN4*, *KCNT1*, *KCNT2*, *KCNU1*
CatSper and two-pore channel	*CATSPER1*, *CATSPER2*, *CATSPER3*, *CATSPER4*, *TPCN1*, *TPCN2*
Cyclic nucleotide-regulated channel	*CNGA1*, *CNGA2*, *CNGA3*, *CNGA4*, *CNGB1*, *CNGB3*, *HCN1*, *HCN2*, *HCN3*, *HCN4*
Inwardly rectifying potassium channel	*KCNJ1*, *KCNJ2*, *KCNJ3*, *KCNJ4*, *KCNJ5*, *KCNJ6*, *KCNJ8*, *KCNJ9*, *KCNJ10*, *KCNJ11*, *KCNJ12*, *KCNJ13*, *KCNJ14*, *KCNJ15*, *KCNJ16*
Transient Receptor Potential channel	*TRPA1*, *TRPC1*, *TRPC2*, *TRPC3*, *TRPC4*, *TRPC5*, *TRPC6*, *TRPC7*, *TRPM1*, *TRPM2*, *TRPM3*, *TRPM4*, *TRPM5*, *TRPM6*, *TRPM7*, *TRPM8*, *MCOLN1*, *MCOLN2*, *MCOLN3*, *PKD2*, *PKD2L1*, *PKD2L2*, *TRPV1*, *TRPV2*, *TRPV3*, *TRPV4*, *TRPV5*, *TRPV6*
Two-P potassium channel	*KCNK1*, *KCNK2*, *KCNK3*, *KCNK4*, *KCNK5*, *KCNK6*, *KCNK7*, *KCNK9*, *KCNK10*, *KCNK12*, *KCNK13*, *KCNK15*, *KCNK16*, *KCNK17*, *KCNK18*
Voltage-gated calcium channel	*CACNA1S*, *CACNA1C*, *CACNA1D*, *CACNA1F*, *CACNA1A*, *CACNA1B*, *CACNA1E*, *CACNA1G*, *CACNA1H*, *CACNA1I*
Voltage-gated potassium channel	*KCNA1*, *KCNA2*, *KCNA3*, *KCNA4*, *KCNA5*, *KCNA6*, *KCNA7*, *KCNA10*, *KCNB1*, *KCNB2*, *KCNC1*, *KCNC2*, *KCNC3*, *KCNC4*, *KCND1*, *KCND2*, *KCND3*, *KCNF1*, *KCNG1*, *KCNG2*, *KCNG3*, *KCNG4*, *KCNQ1*, *KCNQ2*, *KCNQ3*, *KCNQ4*, *KCNQ5*, *KCNV1*, *KCNV2*, *KCNS1*, *KCNS2*, *KCNS3*, *KCNH1*, *KCNH2*, *KCNH3*, *KCNH4*, *KCNH5*, *KCNH6*, *KCNH7*, *KCNH8*
Voltage-gated sodium channel	*SCN1A*, *SCN2A*, *SCN3A*, *SCN4A*, *SCN5A*, *SCN8A*, *SCN9A*, *SCN10A*, *SCN11A*

a Only human gene symbols are listed.

## Materials and Methods

### Genomic Data

The definition of an ion channel gene for human, rat, and mouse was obtained from IUPHAR-DB [31]. We collected the coding sequence for each orthologous ion channel gene from the Reference Sequence (RefSeq) database [32]. In total, 141 orthologous genes from 9 categories of voltage-gated ion channel were involved in this study (Table 1 and Table S1). We built multiple alignments of orthologous sequences based on the peptide sequences with MUSCLE [33]. For each ion channel protein, we considered the residues as transmembrane if they were within the segments annotated as “transmembrane-region” by RefSeq. We also assigned the residues as channel-forming if they were within the region annotated by RefSeq as “BK_channel_a”, “Ion_trans_2”, “Ion_trans”, “KCNQ_channel”, “Kv2channel”, “PKD_channel”, “PLN03192”, “pore-forming domain”, “Potassium_chann”, “Shal-type”, “SK_channel”, “TRP_2”, or “Selectivity filter”.

**Figure 1:**
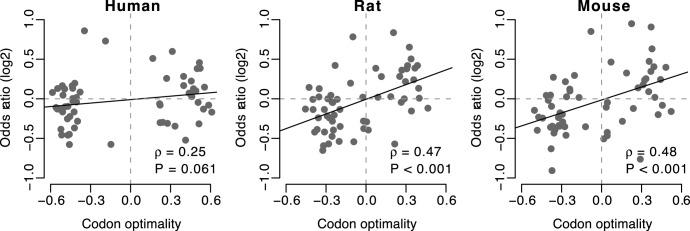
Odds ratio (*OR_1_*) versus codon optimality. With the exception of human, all organisms show a significant correlation between these two quantities. *OR_1_* measures whether the codon is preferred at transmembrane sites, compared to all other codons encoding the same amino acid.

**Table 2:** Example of a 2×2 contingency table for codon GCC in one particular gene in human.

Codon^a^	Transmembrane	Non-transmembrane
GCC	8	5
GCA, GCG, GCT	3	9

a Codon GCC encodes amino acid Ala. The other three non-GCC codons encoding Ala are GCG, GCA, and GCT. The odds ratio of GCC usage between transmembrane and non-transmembrane sites is (8/3)/(5/9) = 4.8 for this contingency table. Because there is one table of GCC per gene, we applied the Mantel–Haenszel procedure to calculate the joint odds ratio of use frequency between transmembrane and non-transmembrane sites for all genes.

### Identifying Optimal Codons

To identify which codons are translationally optimal in each species, we calculated the codon use frequency for each codon for all of the annotated coding sequences in each genome. The effective number of codons (*ENC*) of each gene was also calculated, which measured the overall codon bias of that gene [34]. A lower *ENC* value indicates stronger overall codon bias. We assumed that genes with stronger codon bias were more likely to use optimal codons. We then calculated the Spearman’s rank correlation between the frequency of each codon within each gene and *ENC* of that gene. We defined codons as “optimal” if they showed a statistically significant increase in frequency in the genes with stronger codon bias, which was identified by a significant negative correlation (*P*<0.05 after Benjamini-Hochberg adjustment) between codon frequency and *ENC*. We defined codon optimality as the multiplication product of −1 and the correlation coefficient between codon frequency and *ENC*, calculated separately for each codon.

### Mantel-Haenszel Procedure

For pairs of discrete variables (e.g., optimal vs. non-optimal codons and transmembrane vs. non-transmembrane sites), we stratified the data by gene and synonymous codon family within each gene, and constructed a separate 2×2 contingency table for each stratum. We then combined either the tables for all genes and a given codon family or the tables for all genes and all codon families into an overall analysis, using the Mantel-Haenszel procedure [35]. The null hypothesis in this analysis assumes that the status of the site (e.g., transmembrane or non-transmembrane sites) is independent of the codon type in any given stratum. Because the Mantel-Haenszel procedure yields undefined results on contingency tables whose sum of all four entries is less than 2 (i.e., 0 or 1), we excluded all such tables from the analyses.

## Results

### Optimal Codons are Preferred at Transmembrane Sites

We first assessed whether there was any relationship between a codon’s translational optimality and the same codon’s tendency to be preferentially used at transmembrane sites in voltage-gate ion channel genes. We calculated codon optimality for 59 codons (excluding ATG for Met, TGG for Trp, and three stop codons). Codon optimality measures whether the codon is preferred in genes with strong codon bias (see Materials and Methods), which reflects the translational fidelity of the codon [36], [37]. The codons with higher optimality are more likely to be translated accurately. We also calculated the odds ratio (*OR_1_*) that measures whether the codon is preferred at transmembrane sites compared to all other codons encoding the same amino acid. To control for confounding effects of differing amino acid usage among genes, we computed *OR_1_* by first constructing 2×2 contingency tables of codon usage within each gene (see Table 2 for an example) and then using the Mantel-Haenszel procedure [35] to combine the odds ratios for each individual contingency table into an overall odds ratio. We list the values of codon optimality and *OR_1_* for each codon in Table S2. In all species except for human, we found a significant positive Spearman’s rank correlation between codon optimality and *OR_1_* (*P*<0.001 for rat and mouse, while *P* = 0.061 for human, Figure 1).

The correlation between codon optimality and *OR_1_* reveals that codons with higher optimality are preferred at transmembrane sites in voltage-gate ion channel genes. To determine whether this correlation is consistent across all amino acids or if different amino acids have different trends, we carried out a similar statistical test on each amino acid separately. We inferred a set of optimal codons for each species (see Materials and Methods and Table S3). For each gene, we then constructed separate 2×2 contingency tables for the 18 amino acids encoded by at least two codons (see Table 3 for an example). For each of these 18 amino acids, we calculated a joint odds ratio of optimal codon usage between conserved and non-conserved sites using the Mantel-Haenszel procedure. A joint odds ratio greater than 1 signifies a preference for optimal codons at transmembrane sites (and non-optimal codons at non-transmembrane sites).

**Table 3:** Example of a 2×2 contingency table for amino acid Ala in one particular gene in human.

	Codon^a^	Transmembrane	Non-transmembrane
Optimal	GCC, GCG	9	2
Non-optimal	GCA, GCT	7	7

a Codons GCC and GCG are optimal codons for amino acid Ala in human (see table S3). The odds ratio of optimal codon usage between transmembrane and non-transmembrane sites is (9/7)/(2/7) = 4.5 for this contingency table. Because there is one table of Ala per one gene, we applied the Mantel–Haenszel procedure to calculate the joint odds ratio for all tables of Ala across all genes.

We found that, of a total of 54 association tests, 12 showed a significant preference (before correction for multiple testing) at transmembrane sites for optimal codons, while none showed a significant preference for non-transmembrane optimal codons (Table 4). Interestingly, three amino acids (Ala, Asp, and Val) showed a significant preference for optimal codons at transmembrane residues in all species.

**Table 4:** Odds ratio of optimal codon usage between transmembrane and non-transmembrane sites.

Amino acid	Human^a^	Rat^a^	Mouse^a^
A	1.222**(*)	1.418***	1.177(*)
C	1.217	0.97	0.919
D	1.373(**)	1.571**	1.876***
E	0.946	1.338(*)	1.317
F	0.969	1.005	1.013
G	1.054	1.162	1.038
H	1.168	1	1.131
I	1.107	1.096	0.945
K	0.914	1.308	1.184
L	1.032	1.083	1.08
N	1.072	1.181	1.271
P	0.919	1.285	1.043
Q	1.306	1.338	1.549
R	0.917	1.024	0.941
S	1.067	1.158	1.112
T	0.94	1.301**	1.366**(*)
V	1.161(*)	1.215*(*)	1.249*(*)
Y	1.068	1.216	1.179
Overall	1.061**	1.167***	1.114***

a Significance levels in parentheses disappear after correction for multiple testing.

* *P*<0.05;

** *P*<0.01;

*** *P*<0.001.

For each species, we also used the Mantel-Haenszel procedure to combine all 2×2 contingency tables for all genes and all amino acids into a single overall odds ratio. We found a statistically significant association between optimal codons and transmembrane sites in all species (Table 4).

**Figure 2:**
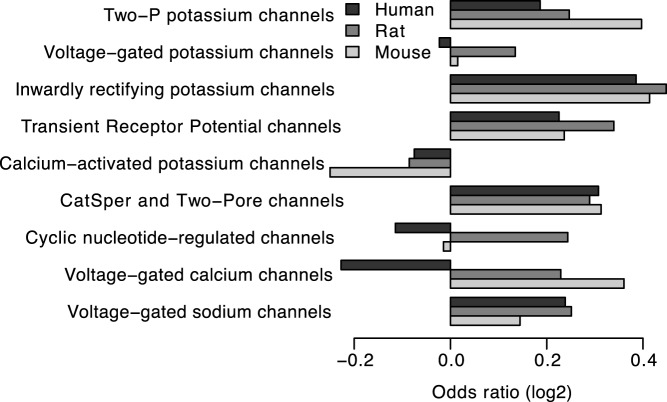
Joint odds ratio of optimal codon usage between transmembrane and non-transmembrane sites for each type of voltage-gated ion channel. The odds ratios were calculated by the Mantel-Haenszel procedure.

To determine if the association between optimal codons and transmembrane sites was affected by the type of voltage-gated ion channel, we calculated the overall odds ratio separately for each channel type listed in Table 1. In all species, the overall odds ratios for two-P potassium channel, inwardly rectifying potassium channel, transient receptor potential channel, CatSper and two-pore channel, and voltage-gated sodium channel were consistently higher than one, while the overall odds ratios for the calcium-activated potassium channel was consistently lower than one in the three species (Figure 2).

### Optimal Codons are Preferred at Channel-forming Sites

Our hypothesis was that, if selection for translational accuracy acts to minimize mistranslation-induced dysfunction of ion channel proteins, then functionally important sites should associate with more optimal codons and vice versa. Thus, we tested for an association between optimal codons and transmembrane sites. Our reasoning was that transmembrane sites are more likely to be involved in channel-forming compared to non-transmembrane sites. An alternative and more direct way is to analyze the sites that are already known to be channel-forming, though the information is still very limited.

**Figure 3:**
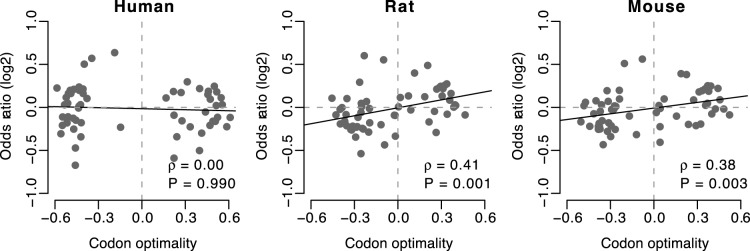
Odds ratio (*OR_2_*) versus codon optimality. With the exception of human, all organisms show a significant correlation between these two quantities. *OR_2_* measures whether the codon is preferred at channel-forming sites, compared to all other codons encoding the same amino acid.

After collecting channel-forming sites by combining data from IUPHAR-DB and RefSeq, we first assessed whether there was any relationship between a codon’s translational optimality and the same codon’s tendency to be preferentially used at channel-forming sites, which was similar to the test we performed for transmembrane/non-transmembrane sites. We calculated the odds ratio (*OR_2_*), which measures whether the codon is preferred at channel-forming sites compared to all other codons encoding the same amino acid. However, we only found a significant positive correlation between codon optimality and *OR_2_* in rat and mouse (Figure 3).

The correlation between codon optimality and *OR_2_* reveals that there is an association between codon usage and residue channel-forming importance, at least in rat and mouse. To determine whether this correlation is consistent across all amino acids or if different amino acids have different trends, we carried out a statistical test on each amino acid separately. For each of the 18 amino acids, we calculated a joint odds ratio of optimal codon usage between channel-forming and non- channel-forming sites using the Mantel-Haenszel procedure. A joint odds ratio greater than one signifies a preference for optimal codons at channel-forming sites (and non-optimal codons at non-channel-forming sites).

We found that 7 of 18 amino acids showed, in at least one species, a significant preference (before correction for multiple testing) for optimal codons at channel-forming residues (Table 5). Unexpectedly, three amino acids (Cys, Glu, and Phe) in human and one amino acid (Cys) in rat showed a significant preference for optimal codons at non-channel-forming sites. Of a total of 54 association tests, 10 showed a significant preference for channel-forming optimal codons, while 4 showed a significant preference for non-channel-forming optimal codons.

For each species, we also used the Mantel-Haenszel procedure to combine all 2×2 contingency tables for all genes and all amino acids into a single overall odds ratio. We only found a statistically significant association between optimal codons and channel-forming sites in rat and mouse (Table 5).

**Table 5:** Odds ratio of optimal codon usage between channel-forming and non-channel-forming sites.

Amino acid	Human^a^	Rat^a^	Mouse^a^
A	0.992	1.208**(*)	1.064
C	0.706**(*)	0.817(*)	0.872
D	1.133	1.196*	1.191(*)
E	0.856(*)	1.002	0.987
F	0.893(*)	1.016	0.948
G	1.017	1.185**	1.160*(*)
H	1.109	1.174	1.133
I	1.022	1.021	1.063
K	1.08	1.118	1.165
L	1.016	1.190***	1.183***
N	0.894	1.06	1.033
P	1.008	1.158(*)	1.061
Q	0.923	1.162	1.139
R	1.003	1.052	0.996
S	1.041	1.235***	1.089
T	0.901	1.096	1.024
V	1.155(*)	1.036	1.072
Y	0.865	0.968	0.874
all	0.985	1.110***	1.067***

a Significance levels in parentheses disappear after correction for multiple testing.

* *P*<0.05;

** *P*<0.01;

*** *P*<0.001.

### Conserved Synonymous Sites in the Transmembrane Region

We assessed whether the synonymous codon sites within transmembrane regions were more conserved than sites outside that region. For this purpose, we only focused on the residues without any non-synonymous substitutions among human, rat, and mouse. Specifically, we only looked into the codon usage pattern for the conserved amino acids. We constructed one 2×2 contingency table for each gene (see Table 6 for an example). The codons without any synonymous substitutions among the three species were assigned as conserved. The joint odds ratio of codon conservation pattern between transmembrane and non-transmembrane regions was 1.101 (*P*<0.001) based on the Mantel-Haenszel procedure, which suggests that synonymous sites are more conserved in transmembrane regions than the sites in non-transmembrane regions.

**Table 6:** Example of a 2×2 contingency table for the conserved/non-conserved codon pattern in one particular gene in human.

	Transmembrane	Non-transmembrane
Without synonymous substitution a	86	61
With synonymous substitution	12	49

a Only the conserved protein residues were involved. The odds ratio of the number of conserved/non-conserved codon sites between transmembrane and non-transmembrane sites is (86/12)/(61/49) = 5.8 for this contingency table. Because there is one table per one gene, we applied the Mantel–Haenszel procedure to calculate the joint odds ratio for all tables across all genes.

**Figure 4:**
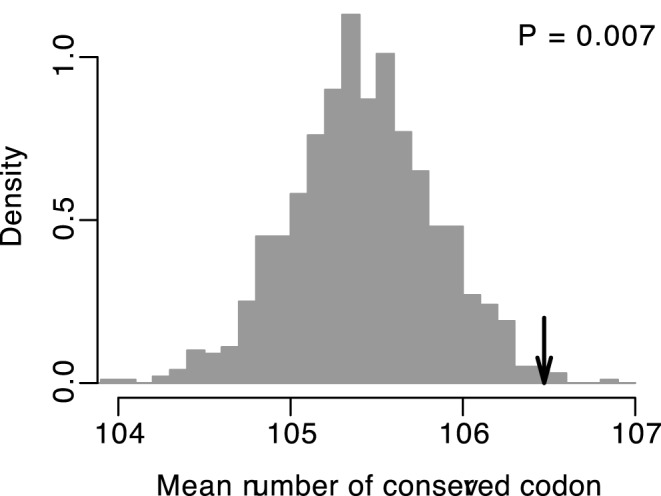
Distribution of the mean number of conserved codons across all the voltage-gated ion channel genes. The black arrows indicate the real mean number of conserved codons across all the voltage-gated ion channel genes. The gray histograms show the random sampling distribution of the same quantity under the null hypothesis, which were generated 1,000 resampled sequences for each gene by randomly reshuffling synonymous codons among sites with identical amino acid. The *P*-value was obtained by one-tailed test.

To avoid the possible bias caused by the difference in amino acid composition of each gene, we conducted a randomization analysis. We first computed the mean number of conserved codons across all voltage-gated ion channel genes. We next generated 1,000 resampled sequences for each gene by randomly reshuffling synonymous codons among sites with identical amino acids. We recalculated the mean number of conserved codons. We then carried out a one-tailed test. Our alternative hypothesis was that the mean number of conserved codons is higher than expected by chance if synonymous sites are more conserved in transmembrane regions than the sites in non-transmembrane regions. We found that, in this case, we could reject the null hypothesis that synonymous sites are not more conserved in transmembrane regions (*P* = 0.007, Figure 4).

## Discussion

Synonymous mutations are the mutations that change the coding sequence of a gene without changing the amino-acid sequence. Because these mutations don’t alter the expressed primary protein sequence, they are also called silent mutations. However, codon usage bias exists in many organisms, which suggests that there is natural selection for the use of particular codons and synonymous sites are not necessarily to be neutral. So far, there are only a limited number of studies reporting the possible effect of silent mutations in ion channel genes. For example, Shah et al. found that one silent polymorphism in potassium inwardly-rectifying channel *KCNJ11* may be related to the disorder hyperinsulinism of infancy. A silent polymorphism at codon 190 was over-represented in the patients who responded well to medical treatment and under-represented in those that required radical surgical intervention [38]. Shah et al. proposed that the changed codon that is not represented by a corresponding anti-codon within the human nuclear tRNA may lead to a decreased rate of expression of the protein [38]. In another study, Richard et al. identified a synonymous mutation in ligand-gated ion channel *CHRNE*, which created a new splice donor site leading to an aberrant splicing of pre-mRNAs and so to their instability. This synonymous mutation was thought to generate a cryptic splice site and be responsible for human congenital myasthenic syndrome [39].

In this study, we focused on the relationship between codon usage bias and residue importance of voltage-gated ion channel proteins in three mammalian genomes. First, we found that optimal codons tend to be associated with transmembrane sites. Second, we analyzed residues involved in channel-forming and found a significant association between optimal codons and channel-forming except in humans. Finally, we assessed whether the synonymous codon sites within transmembrane regions were more conserved than sites outside that region. For this purpose, we only examined the codon usage pattern for the conserved amino acids across human, rat, and mouse species. We found that the conservation level at synonymous sites in transmembrane regions is significantly higher than that in non-transmembrane regions.

Several previous studies have found that selection for translational accuracy should lead to preference of optimal codons at important sites in *Escherichia coli*, yeast, worm, fly, and mammals [24], [25], [26], [27], [30]. Here, we extended this theory to voltage-gated ion channel genes. The synonymous sites within transmembrane or channel-forming regions are not silent. This observation could be caused by selection to reduce mistranslation-induced protein misfolding or mistranslation-induced loss of function. Structurally or functionally important sites prefer synonymous codons with higher translational fidelity to ensure more accurate translation. The transmembrane domain is the key region of ion channel proteins and forms a protein-lined pore through the membrane. Upon activation, the pore becomes accessible to ions, which then pass through. A group of hereditary disorders were found to be associated with ion channel mutations in the transmembrane region [40], which indicates the functional importance of these regions in ion channel proteins. Therefore, synonymous mutations in these regions may cause deleterious mistranslation and thus lead to ion-channel dysfunction, e.g., as recessive mutations lead to loss of function, and dominant mutations lead to change of function [40].

Why do some amino acids show a preference for optimal codon at transmembrane sites while others do not? As we had seen in previous studies [26], [27], there is no consistent pattern among organisms as to which amino acids show a significant signal of translational accuracy selection. There is also no clear pattern related to amino acid biochemistry, such as polarity or volume that would explain either the observed odds ratios or associated *P*-values. Instead, as shown in a previous study [26], the best predictor for *P*-values is amino acid frequency, indicating that much of the variation in the observed results may simply be due to lack of statistical power for rare amino acids. For example, voltage-sensing helices, which are supposed to be functionally sensitive, generally have positively charged arginine. However, we didn’t observe a significant preference of optimal codons for arginine at transmembrane sites from Tables 4 and 5, which may be due to the relatively low amino acid frequency for arginine in this region. Actually, the frequency of arginine at transmembrane sites in each gene is even lower than that of serine and threonine, which are more likely to show up at intracellular region (*P*<10^−10^ by t-test in all three species) (Figure S1). The low frequency of arginine at transmembrane sites may lead to the decreased statistical power of Mantel-Haenszel procedure. Thus we didn’t observe significant preference of optimal codon for arginine at transmembrane sites.

The association between optimal codons and transmembrane sites seems to be affected by the type of voltage-gated ion channel. It is interesting that the overall odds ratios for calcium-activated potassium channels were consistently lower, which may be due to the fact that alternative splicing is very common in the genes coding for calcium-activated potassium channel [41]. Alternative splicing is usually accompanied by strongly increased selection pressure against synonymous mutations, and thus the selection to facilitate alternative splicing at alternatively spliced exons diminishes the effect of selection for translational accuracy [18], [42].

When we directly looked at the sites that are known to be channel-forming, we only found significant signal in rat and mouse (Table 5 and Figure 3), which may be caused by the limitations on quality and quantity of the annotation data. However, when we focus on the sites that are annotated as transmembrane, the signal in human was also weaker than that in rat and mouse (Table 4 and Figure 1). Moreover, the overall odds ratio calculated separately for each channel type showed that there were more channel types with the overall odds ratio <1 in humans compared to rats and mice (Figure 2). These observations may be explainable by the reduced efficacy of natural selection in humans due to the smaller long-term effective population size [43], [44]. The selection at synonymous sites is relatively weaker comparing with the selection at non-synonymous sites because most mutations at synonymous sites are just slightly deleterious [45], [46]. Slightly deleterious mutations in the species with smaller effective population sizes are more likely to be subject to genetic drift and behave as effectively neutral [47]. One the contrary, the species with large effective population sizes have a relatively smaller proportion of effectively neutral mutations [47]. Therefore, the efficiency of purifying selection in removing slightly deleterious mutations is reduced when effective population size is low [44]. Because rodents are reported to have larger population sizes than primates [43], [44], the magnitude of selection at synonymous sites is consequently higher for rodents than primates [48]. Thus, the selection pressure for optimal codons at transmembrane sites is weaker for human than rat and mouse.

We identified translationally optimal codons by correlating codon use frequency with gene codon bias (*ENC*). This method of identifying optimal codons has its limitations in specific cases. For example, if we look at genes in the genomic region with strong mutational bias, the method will yield a set of optimal codons, which just reflects the local GC content [49]. Another problem of this method lies in the translational speed-accuracy tradeoffs. The most rapidly translated codon may not be the most accurately translated or vice versa because speed is determined primarily by the absolute number of tRNA copies in a cell, whereas accuracy depends on the relative abundance of the cognate tRNA compared with competing tRNAs. Nevertheless, we found that the optimal codons for mouse are very similar to one previously published, which identified optimal codons by comparing codon usage bias between highly and lowly expressed genes [26].

We have one implicit assumption in our study that the expected codon composition of transmembrane and non-transmembrane sites within the same gene is equal in the absence of selection. However, if selection causes transversion mutations less often at transmembrane sites than at non- transmembrane sites, the equilibrium codon composition of transmembrane and non-transmembrane sites will differ [50]. In future studies, it would be interesting to test how Morton’s hypothesis can affect the codon usage at important sites within a protein using translational-selection simulation.

This study provides evidence that synonymous sites in voltage-gated ion channel genes are not neutral. Silent mutations should not be neglected because some particular silent mutations at channel-related residues may lead to dysfunction of the ion channel. The fact that synonymous sites are more conserved in transmembrane regions than sites in non-transmembrane regions provides us the possibility to identify the most critical silent sites *in silico* in future studies.

## Supporting Information

Figure S1 **Amino acid frequency at transmembrane sites for arginine (R), serine (S), and threonine (T).** The mean frequencies of S and T are significantly higher than that of R in human, rat, and mouse (*P*<10^−10^ by t-test). The errors indicate the standard errors.
(PDF)

Table S1 **Orthologs of voltage-gated ion channel genes in human, rat, and mouse.**
(PDF)

Table S2 **Codon optimality and corresponding odds ratio (** ***OR_1_*** **) of use frequency between transmembrane and non-transmembrane sites.**
(PDF)

Table S3 **List of optimal codons.**
(PDF)
