# Supplementary material for: Non-Silent Story on Synonymous Sites in Voltage-Gated Ion Channel Genes
Source: PLoS One. 2012 Oct 31;7(10):e48541. doi: 10.1371/journal.pone.0048541 (PMC3485311; doi:10.1371/journal.pone.0048541)
Supplement: Figure S1 — Amino acid frequency at transmembrane sites for arginine (R), serine (S), and threonine (T). The mean frequencies of S and T are significantly higher than that of R in human, rat, and mouse (P<10−10 by t-test). The errors indicate the standard errors. (PDF) [file pone.0048541.s001.pdf]

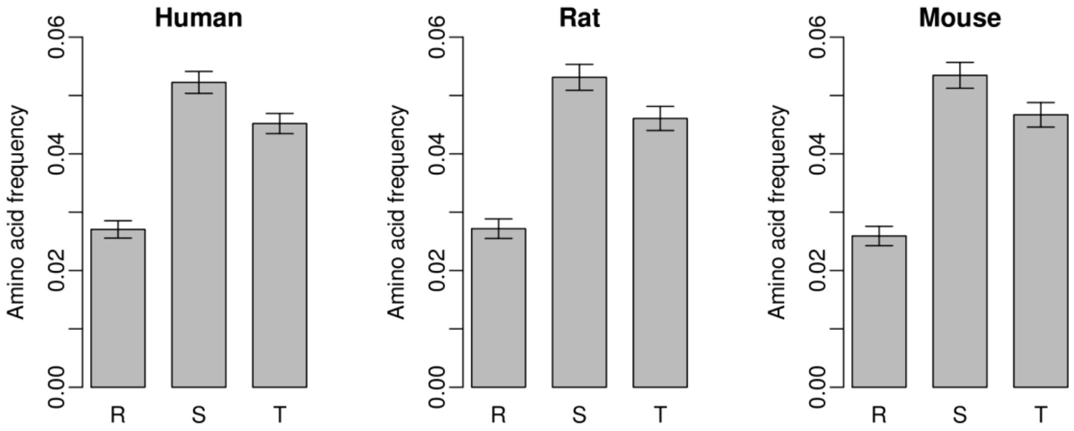

Figure S1. Amino acid frequency at transmembrane sites for arginine (R), serine (S), and threonine (T). The mean frequencies of S and T are significantly higher than that of R in human, rat, and mouse ($P < 10^{-10}$ by t-test). The errors indicate the standard errors.
