# Supplementary material for: Non-Silent Story on Synonymous Sites in Voltage-Gated Ion Channel Genes
Source: PLoS One. 2012 Oct 31;7(10):e48541. doi: 10.1371/journal.pone.0048541 (PMC3485311; doi:10.1371/journal.pone.0048541)
Supplement: Table S1 — Orthologs of voltage-gated ion channel genes in human, rat, and mouse. (PDF) [file pone.0048541.s002.pdf]

Table S1. Orthologs of voltage-gated ion channel genes in human, rat, and mouse

Human gene name	Human nucleotide RefSeq	Rat gene name	Rat nucleotide RefSeq	Mouse gene name	Mouse nucleotide RefSeq
KCNMA1	NM_001014797	*Kcnma1*	NM_031828	*Kcnma1*	NM_010610
KCNN1	NM_002248	*Kcnn1*	NM_019313	*Kcnn1*	NM_032397
KCNN2	NM_170775	*Kcnn2*	NM_019314	*Kcnn2*	NM_080465
KCNN3	NM_002249	*Kcnn3*	NM_019315	*Kcnn3*	NM_080466
KCNN4	NM_002250	*Kcnn4*	NM_023021	*Kcnn4*	NM_008433
KCNT1	NM_020822	*Kcnt1*	NM_021853	*Kcnt1*	NM_175462
KCNT2	NM_198503	*Kcnt2*	NM_198762	*Kcnt2*	NM_001081027
KCNU1	NM_001031836	*Kcnu1*		*Kcnu1*	NM_008432
CATSPER1	NM_053054	*Catsper1*		*Catsper1*	NM_139301
CATSPER2	NM_172097	*Catsper2*	NM_001012220	*Catsper2*	NM_153075
CATSPER3	NM_178019	*Catsper3*	NM_001106101	*Catsper3*	NM_029772
CATSPER4	NM_198137	*Catsper4*		*Catsper4*	NM_177866
TPCN1	NM_017901	*Tpcn1*	NM_139332	*Tpcn1*	NM_145853
TPCN2	NM_139075	*Tpcn2*	NM_001107566	*Tpcn2*	NM_146206
CNGA1	NM_000087	*Cnga1*	NM_053497	*Cnga1*	NM_007723
CNGA2	NM_005140	*Cnga2*	NM_012928	*Cnga2*	NM_007724
CNGA3	NM_001298	*Cnga3*	NM_053495	*Cnga3*	NM_009918
CNGA4	NM_001037329	*Cnga4*	NM_053496	*Cnga4*	NM_001033317
CNGB1	NM_001297	*Cngb1*	NM_031809	*Cngb1*	NM_001195413
CNGB3	NM_019098	*Cngb3*		*Cngb3*	NM_013927
HCN1	NM_021072	*Hcn1*	NM_053375	*Hcn1*	NM_010408
HCN2	NM_001194	*Hcn2*	NM_053684	*Hcn2*	NM_008226
HCN3	NM_020897	*Hcn3*	NM_053685	*Hcn3*	NM_008227
HCN4	NM_005477	*Hcn4*	NM_021658	*Hcn4*	NM_001081192
KCNJ1	NM_000220	*Kcnj1*	NM_017023	*Kcnj1*	NM_019659
KCNJ2	NM_000891	*Kcnj2*	NM_017296	*Kcnj2*	NM_008425
KCNJ12	NM_021012	*Kcnj12*	NM_053981	*Kcnj12*	NM_010603
KCNJ4	NM_152868	*Kcnj4*	NM_053870	*Kcnj4*	NM_008427
KCNJ14	NM_170720	*Kcnj14*	NM_170718	*Kcnj14*	NM_145963
KCNJ3	NM_002239	*Kcnj3*	NM_031610	*Kcnj3*	NM_008426
KCNJ6	NM_002240	*Kcnj6*	NM_013192	*Kcnj6*	NM_010606
KCNJ9	NM_004983	*Kcnj9*	NM_053834	*Kcnj9*	NM_008429
KCNJ5	NM_000890	*Kcnj5*	NM_017297	*Kcnj5*	NM_010605
KCNJ10	NM_002241	*Kcnj10*	NM_031602	*Kcnj10*	NM_001039484
KCNJ15	NM_170736	*Kcnj15*	NM_133321	*Kcnj15*	NM_001039057
KCNJ16	NM_018658	*Kcnj16*	NM_053314	*Kcnj16*	NM_010604
KCNJ8	NM_004982	*Kcnj8*	NM_017099	*Kcnj8*	NM_008428
KCNJ11	NM_000525	*Kcnj11*	NM_031358	*Kcnj11*	NM_010602
KCNJ13	NM_002242	*Kcnj13*	NM_053608	*Kcnj13*	NM_001110227

TRPA1	NM_007332	*Trpa1*	NM_207608	*Trpa1*	NM_177781
TRPC1	NM_003304	*Trpc1*	NM_053558	*Trpc1*	NM_011643
TRPC2		*Trpc2*		*Trpc2*	NM_011644
TRPC3	NM_003305	*Trpc3*	NM_021771	*Trpc3*	NM_019510
TRPC4	NM_016179	*Trpc4*	NM_080396	*Trpc4*	NM_016984
TRPC5	NM_012471	*Trpc5*	NM_080898	*Trpc5*	NM_009428
TRPC6	NM_004621	*Trpc6*	NM_053559	*Trpc6*	NM_013838
TRPC7	NM_020389	*Trpc7*	NM_001191691	*Trpc7*	NM_012035
TRPM1	NM_002420	*Trpm1*	NM_001037734	*Trpm1*	NM_018752
TRPM2	NM_003307	*Trpm2*	NM_001011559	*Trpm2*	NM_138301
TRPM3	NM_020952	*Trpm3*	XM_219902	*Trpm3*	NM_001035239
TRPM4	NM_017636	*Trpm4*	NM_001136229	*Trpm4*	NM_175130
TRPM5	NM_014555	*Trpm5*	NM_001191896	*Trpm5*	NM_020277
TRPM6	NM_017662	*Trpm6*	XM_219747	*Trpm6*	NM_153417
TRPM7	NM_017672	*Trpm7*	XM_001056331	*Trpm7*	NM_021450
TRPM8	NM_024080	*Trpm8*	NM_134371	*Trpm8*	NM_134252
MCOLN1	NM_020533	*Mcoln1*	NM_001105903	*Mcoln1*	NM_053177
MCOLN2	NM_153259	*Mcoln2*	NM_001039005	*Mcoln2*	NM_001005846
MCOLN3	NM_018298	*Mcoln3*	NM_001012059	*Mcoln3*	NM_134160
PKD2	NM_000297	*Pkd2*	NM_001191934	*Pkd2*	NM_008861
PKD2L1	NM_016112	*Pkd2l1*	NM_001106352	*Pkd2l1*	NM_181422
PKD2L2	NM_014386	*Pkd2l2*	NM_001106156	*Pkd2l2*	NM_016927
TRPV1	NM_018727	*Trpv1*	NM_031982	*Trpv1*	NM_001001445
TRPV2	NM_016113	*Trpv2*	NM_017207	*Trpv2*	NM_011706
TRPV3	NM_145068	*Trpv3*	NM_001025757	*Trpv3*	NM_145099
TRPV4	NM_021625	*Trpv4*	NM_023970	*Trpv4*	NM_022017
TRPV5	NM_019841	*Trpv5*	NM_053787	*Trpv5*	NM_001007572
TRPV6	NM_018646	*Trpv6*	NM_053686	*Trpv6*	NM_022413
KCNK1	NM_002245	*Kcnk1*	NM_021688	*Kcnk1*	NM_008430
KCNK2	NM_001017424	*Kcnk2*	NM_172042	*Kcnk2*	NM_010607
KCNK3	NM_002246	*Kcnk3*	NM_033376	*Kcnk3*	NM_010608
KCNK4	NM_033310	*Kcnk4*	NM_053804	*Kcnk4*	NM_008431
KCNK5	NM_003740	*Kcnk5*	NM_001039516	*Kcnk5*	NM_021542
KCNK6	NM_004823	*Kcnk6*	NM_053806	*Kcnk6*	NM_001033525
KCNK7	NM_033347	*RGD1565025*		*Kcnk7*	NM_010609
KCNK9	NM_016601	*Kcnk9*	NM_053405	*Kcnk9*	NM_001033876
KCNK10	NM_021161	*Kcnk10*	NM_023096	*Kcnk10*	NM_029911
KCNK12	NM_022055	*Kcnk12*	NM_022292	*Kcnk12*	NM_199251
KCNK13	NM_022054	*Kcnk13*	NM_022293	*Kcnk13*	NM_146037
KCNK15	NM_022358	*Kcnk15*	NM_130813	*Kcnk15*	NM_001030292
KCNK16	NM_032115	*Kcnk16*	NM_001109520	*Kcnk16*	NM_029006
KCNK17	NM_031460				
KCNK18	NM_181840	*Kcnk18*	NM_001003820	*Kcnk18*	NM_207261

CACNA1S	NM_000069	*Cacna1s*	NM_053873	*Cacna1s*	NM_001081023
CACNA1C	NM_000719	*Cacna1c*	NM_012517	*Cacna1c*	NM_009781
CACNA1D	NM_000720	*Cacna1d*	NM_017298	*Cacna1d*	NM_028981
CACNA1F	NM_005183	*Cacna1f*	NM_053701	*Cacna1f*	NM_019582
CACNA1A	NM_023035	*Cacna1a*	NM_012918	*Cacna1a*	NM_007578
CACNA1B	NM_000718	*Cacna1b*	NM_147141	*Cacna1b*	NM_001042528
CACNA1E	NM_000721	*Cacna1e*	NM_019294	*Cacna1e*	NM_009782
CACNA1G	NM_198387	*Cacna1g*	NM_031601	*Cacna1g*	NM_009783
CACNA1H	NM_021098	*Cacna1h*	NM_153814	*Cacna1h*	NM_021415
CACNA1I	NM_001003406	*Cacna1i*	NM_020084	*Cacna1i*	NM_001044308
KCNA1	NM_000217	*Kcna1*	NM_173095	*Kcna1*	NM_010595
KCNA2	NM_004974	*Kcna2*	NM_012970	*Kcna2*	NM_008417
KCNA3	NM_002232	*Kcna3*	NM_019270	*Kcna3*	NM_008418
KCNA4	NM_002233	*Kcna4*	NM_012971	*Kcna4*	NM_021275
KCNA5	NM_002234	*Kcna5*	NM_012972	*Kcna5*	NM_145983
KCNA6	NM_002235	*Kcna6*	NM_023954	*Kcna6*	NM_013568
KCNA7	NM_031886	*Kcna7*	NM_001108914	*Kcna7*	NM_010596
KCNA10	NM_005549	*Kcna10*	NM_001191713	*Kcna10*	NM_001074609
KCNB1	NM_004975	*Kcnb1*	NM_013186	*Kcnb1*	NM_008420
KCNB2	NM_004770	*Kcnb2*	NM_054000	*Kcnb2*	NM_001098528
KCNC1	NM_004976	*Kcnc1*	NM_012856	*Kcnc1*	NM_008421
KCNC2	NM_153748	*Kcnc2*	NM_139217	*Kcnc2*	NM_001025581
KCNC3	NM_004977	*Kcnc3*	NM_053997	*Kcnc3*	NM_008422
KCNC4	NM_004978	*Kcnc4*	NM_001122776	*Kcnc4*	NM_145922
KCND1	NM_004979	*Kcnd1*	NM_001105748	*Kcnd1*	NM_008423
KCND2	NM_012281	*Kcnd2*	NM_031730	*Kcnd2*	NM_019697
KCND3	NM_172198	*Kcnd3*	NM_031739	*Kcnd3*	NM_019931
KCNF1	NM_002236	*Kcnf1*	NM_001169104	*Kcnf1*	NM_201531
KCNG1	NM_002237	*Kcng1*	NM_001106545	*Kcng1*	NM_001081134
KCNG2	NM_012283	*Kcng2*	NM_001107372	*Kcng2*	NM_001190373
KCNG3	NM_133329	*Kcng3*	NM_001033957	*Kcng3*	NM_153512
KCNG4	NM_172347	*Kcng4*	NM_001107435	*Kcng4*	NM_025734
KCNQ1	NM_000218	*Kcnq1*	NM_032073	*Kcnq1*	NM_008434
KCNQ2	NM_172107	*Kcnq2*	NM_133322	*Kcnq2*	NM_010611
KCNQ3	NM_004519	*Kcnq3*	NM_031597	*Kcnq3*	NM_152923
KCNQ4	NM_004700	*Kcnq4*	XM_233477	*Kcnq4*	NM_001081142
KCNQ5	NM_019842	*Kcnq5l*	XM_001071249	*Kcnq5*	NM_023872
KCNV1	NM_014379	*Kcnv1*	NM_021697	*Kcnv1*	NM_026200
KCNV2	NM_133497	*Kcnv2*	NM_001106370	*Kcnv2*	NM_183179
KCNS1	NM_002251	*Kcns1*	NM_053954	*Kcns1*	NM_008435
KCNS2	NM_020697	*Kcns2*	NM_023966	*Kcns2*	NM_181317
KCNS3	NM_002252	*Kcns3*	NM_031778	*Kcns3*	NM_173417
KCNH1	NM_172362	*Kcnh1*	NM_031742	*Kcnh1*	NM_010600

KCNH5	NM_172375	*Kcnh5*	NM_133610	*Kcnh5*	NM_172805
KCNH2	NM_172056	*Kcnh2*	NM_053949	*Kcnh2*	NM_013569
KCNH6	NM_173092	*Kcnh6*	NM_053937	*Kcnh6*	NM_001037712
KCNH7	NM_173162	*Kcnh7*	NM_131912	*Kcnh7*	NM_133207
KCNH8	NM_144633	*Kcnh8*	NM_145095	*Kcnh8*	NM_001031811
KCNH3	NM_012284	*Kcnh3*	NM_017108	*Kcnh3*	NM_010601
KCNH4	NM_012285	*Kcnh4*	NM_053630	*Kcnh4*	NM_001081194
SCN1A	NM_006920	*Scn1a*	NM_030875	*Scn1a*	NM_018733
SCN2A	NM_001040142	*Scn2a1*	NM_012647	*Scn2a1*	NM_001099298
SCN3A	NM_006922	*Scn3a*	NM_013119	*Scn3a*	NM_018732
SCN4A	NM_000334	*Scn4a*	NM_013178	*Scn4a*	NM_133199
SCN5A	NM_001099404	*Scn5a*	NM_013125	*Scn5a*	NM_021544
SCN8A	NM_014191	*Scn8a*	NM_019266	*Scn8a*	NM_001077499
SCN9A	NM_002977	*Scn9a*	NM_133289	*Scn9a*	NM_018852
SCN10A	NM_006514	*Scn10a*	NM_017247	*Scn10a*	NM_009134
SCN11A	NM_014139	*Scn11a*	NM_019265	*Scn11a*	NM_011887
