# Supplementary material for: Non-Silent Story on Synonymous Sites in Voltage-Gated Ion Channel Genes
Source: PLoS One. 2012 Oct 31;7(10):e48541. doi: 10.1371/journal.pone.0048541 (PMC3485311; doi:10.1371/journal.pone.0048541)
Supplement: Table S2 — Codon optimality and corresponding odds ratio ( OR1 ) of use frequency between transmembrane and non-transmembrane sites. (PDF) [file pone.0048541.s003.pdf]

Table S2. Codon optimality and corresponding odds ratio (OR_1) of use frequency between transmembrane and non-transmembrane sites

		Human		Rat		Mouse	
Amino acid	codon	Codon optimality	OR_1	Codon optimality	OR_1	Codon optimality	OR_1
A	GCA	-0.470	0.727	-0.281	0.674	-0.357	0.741
A	GCC	0.500	1.303	0.338	1.418	0.393	1.324
A	GCG	0.222	0.811	-0.017	0.770	0.043	0.724
A	GCT	-0.434	1.002	-0.250	0.968	-0.263	1.018
C	TGC	0.398	1.217	0.264	0.970	0.239	0.919
C	TGT	-0.398	0.822	-0.264	1.031	-0.239	1.088
D	GAC	0.517	1.373	0.325	1.571	0.372	1.876
D	GAT	-0.517	0.728	-0.325	0.636	-0.372	0.533
E	GAA	-0.586	1.057	-0.386	0.747	-0.483	0.759
E	GAG	0.586	0.946	0.386	1.338	0.483	1.317
F	TTC	0.507	0.969	0.399	1.005	0.400	1.013
F	TTT	-0.507	1.032	-0.399	0.995	-0.400	0.987
G	GGA	-0.510	0.892	-0.313	0.911	-0.376	0.936
G	GGC	0.472	1.092	0.296	1.162	0.354	1.065
G	GGG	0.222	0.946	0.004	0.817	0.044	0.968
G	GGT	-0.379	1.061	-0.116	1.146	-0.169	1.012
H	CAC	0.456	1.168	0.287	1.000	0.312	1.131
H	CAT	-0.456	0.856	-0.287	1.000	-0.312	0.884
I	ATA	-0.459	0.670	-0.320	0.663	-0.383	0.779
I	ATC	0.555	1.107	0.395	1.096	0.456	0.945
I	ATT	-0.495	1.090	-0.352	1.093	-0.362	1.208
K	AAA	-0.547	1.094	-0.367	0.764	-0.443	0.844
K	AAG	0.547	0.914	0.367	1.308	0.443	1.184
L	CTA	-0.424	0.892	-0.194	0.785	-0.267	0.727
L	CTC	0.271	1.196	0.115	1.215	0.188	1.253
L	CTG	0.610	0.890	0.464	0.921	0.522	0.895
L	CTT	-0.534	0.914	-0.397	0.910	-0.415	1.030
L	TTA	-0.559	0.926	-0.428	1.105	-0.504	0.904
L	TTG	-0.512	1.122	-0.354	1.017	-0.383	1.033
N	AAC	0.490	1.072	0.358	1.181	0.370	1.271
N	AAT	-0.490	0.933	-0.358	0.846	-0.370	0.787
P	CCA	-0.419	1.146	-0.202	0.689	-0.294	0.873
P	CCC	0.459	1.020	0.268	1.285	0.333	1.192
P	CCG	0.241	0.814	-0.024	1.014	0.043	0.745
P	CCT	-0.406	0.973	-0.216	0.995	-0.237	1.072
Q	CAA	-0.523	0.766	-0.312	0.748	-0.378	0.646
Q	CAG	0.523	1.306	0.312	1.338	0.378	1.549
R	AGA	-0.459	0.733	-0.255	0.720	-0.350	0.762
R	AGG	-0.147	0.671	-0.092	0.686	-0.120	0.783
R	CGA	-0.346	1.814	-0.232	1.340	-0.203	1.227
R	CGC	0.399	1.123	0.185	1.019	0.274	1.101
R	CGG	0.334	0.787	0.089	1.014	0.156	0.821
R	CGT	-0.188	1.659	-0.099	1.720	-0.080	1.804
S	AGC	0.413	0.696	0.215	0.673	0.291	0.589
S	AGT	-0.421	0.775	-0.291	0.860	-0.298	0.799
S	TCA	-0.406	1.012	-0.239	0.867	-0.315	0.787
S	TCC	0.313	1.514	0.206	1.785	0.225	1.931
S	TCG	0.218	0.957	0.017	0.761	0.017	0.704
S	TCT	-0.434	1.096	-0.255	0.970	-0.271	1.162
T	ACA	-0.433	0.980	-0.196	0.911	-0.276	0.783
T	ACC	0.439	1.051	0.305	1.301	0.357	1.388
T	ACG	0.277	0.807	-0.019	0.823	0.035	0.929
T	ACT	-0.437	1.121	-0.275	0.846	-0.292	0.817
V	GTA	-0.474	0.839	-0.245	0.761	-0.321	0.792
V	GTC	0.167	1.424	0.073	1.307	0.071	1.379
V	GTG	0.513	0.803	0.340	0.898	0.425	0.871
V	GTT	-0.549	0.914	-0.455	0.914	-0.472	0.851
Y	TAC	0.470	1.068	0.253	1.216	0.323	1.179
Y	TAT	-0.470	0.936	-0.253	0.822	-0.323	0.848
