# Supplementary material for: Non-Silent Story on Synonymous Sites in Voltage-Gated Ion Channel Genes
Source: PLoS One. 2012 Oct 31;7(10):e48541. doi: 10.1371/journal.pone.0048541 (PMC3485311; doi:10.1371/journal.pone.0048541)
Supplement: Table S3 — List of optimal codons. (PDF) [file pone.0048541.s004.pdf]

Table S3. List of optimal codons

Amino acid	Human	Rat	Mouse
A	GCC, GCG	GCC	GCC, GCG
C	TGC	TGC	TGC
D	GAC	GAC	GAC
E	GAG	GAG	GAG
F	TTC	TTC	TTC
G	GGC, GGG	GGC	GGC, GGG
H	CAC	CAC	CAC
I	ATC	ATC	ATC
K	AAG	AAG	AAG
L	CTC, CTG	CTC, CTG	CTC, CTG
N	AAC	AAC	AAC
P	CCC, CCG	CCC	CCC, CCG
Q	CAG	CAG	CAG
R	CGC, CGG	CGC, CGG	CGC, CGG
S	AGC, TCC, TCG	AGC, TCC, TCG	AGC, TCC, TCG
T	ACC, ACG	ACC	ACC, ACG
V	GTC, GTG	GTC, GTG	GTC, GTG
Y	TAC	TAC	TAC
